# Correction: Lungu et al. Molecular Characterisation of Antimicrobial Resistance in *E. coli* Isolates from Piglets in the West Region of Romania. *Antibiotics* 2023, *12*, 1544

**DOI:** 10.3390/antibiotics13050412

**Published:** 2024-04-30

**Authors:** Bianca Cornelia Lungu, Ioan Hutu, Paul Andrew Barrow

**Affiliations:** 1 Horia Cernescu Research Unit, Faculty of Veterinary Medicine, University of Life Sciences King Michael I, Calea Aradului 119, 300645 Timisoara, Romania; bianca.lungu@fmvt.ro; 2 School of Veterinary Medicine, University of Surrey, Daphne Jackson Rd., Guildford, Surrey GU2 7AL, UK

The authors Bianca Cornelia Lungu and Ioan Hutu did not state contributed equally. The corrected Author Contributions statement appears here. The authors state that the scientific conclusions are unaffected. This correction was approved by the Academic Editor. The original publication [1] has also been updated.

Bianca Cornelia Lungu ^1,†^, Ioan Hutu ^1,^*^,†^ and Paul Andrew Barrow ^1,2,^*

^†^ These authors contributed equally to this work.

## Text Correction

There was an error in the original publication. Some ratios from paragraphs Section 2.2 and Section 2.3 were calculated incorrectly.

A correction has been made to content in Section 2.2 and Section 2.3.

### 2.2. Prevalence of Resistance Genes

The prevalence of the resistance genes ampC, blaZ, blaTEM and tetK against the tested antibiotics, detected in susceptible and resistant isolates, are presented in Table 2 [1]. The prevalence of resistance genes (RG+ and SG+) in resistant (R) and susceptible (S) isolates, the penetrance of the genes (P%) and diagnostic odds ratios of positive phenotypic resistance (DOR) for *E. coli* in growing pigs are shown. They show that 62.8% (470/748) of the 76 isolates tested for 13 antibiotics and possessing the ampC, blaZ, blaTEM and tetK genes (RG+) actually showed the resistant phenotype (R) in AST identified using MicroScan Walk Away. More importantly, 61.3% (426/695) of the isolates possessing the genes studied (SG+) actually showed the susceptible phenotype (S) in AST. The genes studied were possessed by 62.1% (896/1443) of R&S isolates. Only 37.2% (278/748) of isolates did not possess the genes studied (RG−) in the AST resistant phenotype (R) and 38.7% (269/695) of isolates did not possess the genes studied (SG−) in the susceptible phenotype (S). Some isolates showed the multi-resistance genes ampC and blaTEM (resistance to cefazolin, cefepime, cefotaxime, cefoxitin, ceftazidime, cefuroxime) and other isolates showed multi-resistance genes ampC, blaZ and blaTEM (resistance to amoxicillin/clavulanate and ampicillin).

### 2.3. Microbiological Antibiotic Susceptibility Testing (AST) by Resistance Genes

The prevalence of resistance genes for phenotypic resistance and their susceptibility to the antibiotics tested are presented in Table 3. The prevalence of ampC was 97.0%, for blaZ it was 96.0%, for blaTEM 32.9% and for tetK 59.3%.

The authors state that the scientific conclusions are unaffected. This correction was approved by the Academic Editor. The original publication has also been updated.
